# Non-Invasive Measurement of Hepatic Fibrosis by Transient Elastography: A Narrative Review

**DOI:** 10.3390/v15081730

**Published:** 2023-08-13

**Authors:** Luca Rinaldi, Chiara Giorgione, Andrea Mormone, Francesca Esposito, Michele Rinaldi, Massimiliano Berretta, Raffaele Marfella, Ciro Romano

**Affiliations:** 1 Department of Advanced Medical and Surgical Sciences, “Luigi Vanvitelli” University of Campania, 80131 Naples, Italy; luca.rinaldi@unicampania.it (L.R.); raffaele.marfella@unicampania.it (R.M.); 2 Department of Neurosciences, Reproductive and Odontostomatological Sciences, “Federico II” University of Naples, 80131 Naples, Italy; michele.rinaldi2@unina.it; 3 Department of Clinical and Experimental Medicine, University of Messina, 98121 Messina, Italy; mberretta@unime.it

**Keywords:** transient elastography, FibroScan, liver stiffness, viral hepatitis, non-alcoholic fatty liver disease, portal hypertension

## Abstract

Transient elastography by FibroScan^®^ (Echosens, Paris, France) is a non-invasive method that can provide a reliable measurement of liver fibrosis through the evaluation of liver stiffness. Despite its limitations and risks, liver biopsy has thus far been the only procedure able to provide data to quantify fibrosis. Scientific evidence and clinical practice have made it possible to use FibroScan^®^ in the diagnostic work-up of several liver diseases to monitor patients’ long-term treatment response and for complication prevention. For these reasons, this procedure is widely used in clinical practice and is still being investigated for further applications. The aim of this narrative review is to provide a comprehensive overview of the main applications of transient elastography in the current clinical practice.

## 1. Introduction

Transient elastography (TE) by FibroScan^®^ (Echosens, Paris, France) is a non-invasive method dedicated to the measurement of liver fibrosis [1]. The device has a probe capable of emitting ultrasound and an elastic wave that passes through the liver parenchyma and measures liver stiffness (LS) [2]. The software on board the machine processes the data of the crossing speed of the elastic wave in the liver and provides a numerical value expressed in Kilopascals for each measurement. The median of 10 measurements represents the result of the examination [2,3].

Over the years, several studies have been carried out to compare histological data and the LS obtained by FibroScan^®^ [3]. These studies have allowed for the elaboration of cut-offs in KpA corresponding to the different degrees of fibrosis (Metavir); despite the presence of some discrepancies, the histological and physical data of the LS have been proven to be sufficiently comparable [4]. Overall, the cut-offs have shown that, in different liver diseases, TE provides a satisfactory diagnostic accuracy for the identification of the degree of fibrosis [5]. Since 2005, TE has thus entered clinical practice in hepatology, especially for the evaluation of patients with chronic viral hepatitis [6,7]. In 2015, this procedure was also used to select hepatitis C patients with severe fibrosis and cirrhosis for treatment with new antiviral therapies [8].

**Figure 1:**
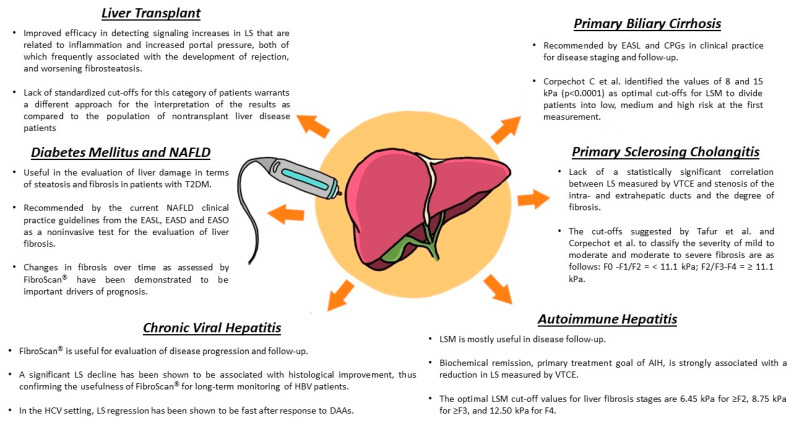
Main FibroScan^®^ applications in hepatology. Summary of the most relevant features in different liver conditions. Detailed discussion is provided in the specific disease paragraphs.

Moreover, the technology has made use of a new software that can provide an estimate of the amount of liver fat (controlled attenuation parameter—CAP) which has extended the use of FibroScan^®^ in the setting of NAFLD [9,10]. The dual function of the device has, therefore, allowed for estimating fibrosis and hepatic steatosis, and to evaluate the severity of a patient at the first visit (Figure 1). Further studies were devoted to the ability of TE to monitor patients undergoing therapy (antiviral or dietary); in this case, the role of the inflammatory component suggested an interpretation of the data based not on cut-offs, but on delta-KpA [11]. From this perspective, TE has also been useful in identifying patients who are at a higher risk of developing complications of cirrhosis or who have a higher risk related to the metabolic component [7]. To date, FibroScan^®^ is now present in all of the main specialized hepatology centres. Therefore, it is included in the diagnostic algorithm of liver diseases, as it has been shown to be a non-invasive method of reliable diagnostic value [12]. The purpose of this review was to provide an overview of the main applications of FibroScan^®^, apart from the viral hepatitis field.

## 2. Technical Notes

The quality of TE is based on the congruence of 10 measurements. An interquartile range (IQR) <30% represents a reliable test threshold according to the manufacturer’s instructions. Boursier further confirmed this concept by proposing that an IQR/median (M) threshold ≤0.10 is a very reliable result, based on a multi-centre study that evaluated liver biopsy as the gold standard [13]. TE is a reproducible test, and it is important to evaluate inter-operator variability, especially in the context of a scientific study. In theory, two experienced operators should perform examinations with low variability and high concordance [14,15]. However, there are some elements to consider when carrying out the exam. Some points of the liver parenchyma, especially those closer to the capsule or vascular structures, have an altered LS; moreover, failure to comply with fasting can also lead to an overestimation of LS. In this case, the TE result may not be correct despite having a low IQR [16]. In addition, a different physical conformation of the rib cage, the presence of excessive fat, or a different hepatic volumetry are elements that may affect the point in the intercostal space, and the distance from the anterior or middle axillary line, at which to lay the probe for the 10 measurements [17]. In this regard, the use of the ultrasound probe to identify an optimal measurement point is a modality that can offer advantages [18].

The latest generation of FibroScan^®^ models have a standard ultrasound probe on board that allows for the visualization of the liver segment on which to perform the measurement [19]. An additional parameter to consider to maintain a high quality of the examination is the elastogram that shows up on the screen for each measurement. Mendes et al. [20] described three categories based on the length of the graphic representation and shear wave dispersal (level of parallelism displayed in the elastogram). A comparison with biopsy data showed that the diagnostic accuracy of TE was significantly greater when based on the quality of each individual measurement.

Another important element that can affect the quality of the examination is the subcutaneous fat related to the condition of being overweight/obesity. In this case, the excess fat thickness increases the distance between the skin surface and the liver capsule. In such circumstances, clinical studies have shown that the use of the XL probe improves the accuracy of LS in difficult patients with BMI > 28 [21,22]. Because of these issues, training in the use of FibroScan^®^ should always include knowledge of all cofactors that can impact performance. Inter-operator variability and low IQR/M should be associated with the other elements described above.

## 3. Transient Elastography in Chronic Viral Hepatitis

Evaluation of the progression of liver fibrosis and cirrhosis is of paramount importance in the management and prognosis of patients with viral hepatitis. Non-invasive methods aim to reduce but not replace liver biopsy. In contrast to chronic hepatitis C (CHC), typical features of chronic hepatitis B (CHB), such as a macronodular histological pattern of cirrhosis and fluctuating necroinflammatory activity, may affect the accuracy of TE with FibroScan^®^ and, therefore, different cut-offs for fibrosis and cirrhosis may be needed [23]. Significant fibrosis (>F2) was usually present in 54% of HCV patients and 42% of HBV patients (*p* = 0.005), while fibrosis >F3 was detected in 24% of HCV subjects and 17% of HBV patients (*p* = 0.048). Very often, HCV patients are also affected by fatty liver disease (23% vs. 11% *p* < 0.001) [24].

Fibrosis progression is an early estimate of the severity of CHB [23]. Since 2003, the FibroTest (FT) [25] and TE [26] have been widely validated in CHB as markers of liver fibrosis as well as prognostic and mortality indices [27,28], partly replacing liver biopsy. Numerous studies have demonstrated that FT and TE are comparable to a biopsy. However, due to the very low incidence of serious complications related to CHB within five years [29], a longer follow-up is needed to evaluate their efficacy. Poynard et al. [30] evaluated FT and TE as markers of the onset of liver cirrhosis associated or not with esophageal varices, but above all, they evaluated the dynamics of fibrosis in patients with sustained virological response (SVR). In this study, which included 1312 patients, esophageal varices occurred in 14 patients after 10 years (1.7% incidence), and serious complications, including hepatocellular carcinoma (HCC), occurred in 25 patients (3.7% incidence) in the same time frame. Despite SVR, the overall incidence of cirrhosis was high, with a residual risk of HCC of 5.7%. The performance of LS in detecting significant fibrosis, however, remains inferior to that in detecting cirrhosis [31]. It has been reported that exacerbations related to hepatitis can influence LS values, which is why, when carrying out the measurement, it is advisable to keep the alanine aminotransferase (ALT) values in mind [32]. Indeed, some authors believe that it is advisable to make a correction, and the European Association for the Study of the Liver (EASL) suggests not using vibration-controlled transient elastography (VCTE) with transaminase levels >10× the upper limit of normal [33]. A study including a large number of participants also demonstrated that LS could be supplemental to HBV DNA for following inactive carrier patients or for assessing disease or fibrosis activity which may even be worthy of a follow-up biopsy [34,35]. It was found that, in antiviral treatment-naïve patients with normal bilirubin and stable chronic hepatitis B (CHB), LS values of 10.6 kPa and 17.0 kPa could be used as cut-offs to exclude and confirm the diagnosis of cirrhosis, respectively. For patients with a LS between 10.6 kPa and 17.0 kPa, ALT cut-offs lower or higher than twice the normal value could be evaluated to confirm or not the diagnosis of advanced fibrosis and initiate antiviral treatment [36]. Wong et al. [37] demonstrated that the liver stiffness measurement (LSM)-HCC score based on age, serum albumin levels, and HBV-DNA is particularly accurate for predicting HCC in patients with CHB. In a study carried out on 600 subjects with CHB, it was found that the patient prognosis worsened as LS increased; the overall five-year survival rate was 97.1% in patients with a LS < 9 kPa and 61.5% in patients with a LS > 20 kPa [38]. The potential of LS for predicting clinical outcomes, therefore, appears to be greater than that of liver biopsy, as LS is able to quantify ongoing pathophysiological events, as opposed to liver biopsy. A recent work has, in fact, demonstrated that baseline LS rather than the histological study of fibrosis was independently predictive of the development of HCC in patients with CHB at the beginning of antiviral treatment [39]. Furthermore, in HBV patients, their HBeAg status had no impact on the diagnostic performance of TE in determining fibrosis status F2 [24]. The evaluation of liver fibrosis in patients with chronic viral hepatitis is important not only to assess the progression and disease prognosis but also to determine the effects of the therapeutic approach. Therefore, attempts have been made to evaluate the effectiveness of various non-invasive tools, such as TE with FibroScan^®^ and other scores, including a fibrosis index based on four factors (FIB-4) and the aspartate aminotransferase-to-platelet ratio index (APRI), to estimate the therapeutic success of some antivirals. Direct anti-viral agents (DAAs) have been used to eradicate HCV. It has been shown that the regression of liver fibrosis using antivirals in patients with chronic hepatitis C (CHC) could be related to reduction in hepatic necroinflammation. Elsharkawy et al. [40] carried out a study which investigated the aforementioned non-invasive methods in patients with HCV and who were treated with sofosbuvir. They found that, following a therapy-driven SVR at 12 months, improvements were obtained in such parameters as platelet counts, transaminase levels, FIB-4, APRI, and liver LS assessed by FibroScan^®^. These improvements were evident in patients who had a baseline LS value higher than 12.5 kPa. However, care must be taken in the use of FIB-4 to exclude liver cirrhosis, as many components used in its calculation, such as age and platelet variations due to other extrahepatic causes, could influence clinical and therapeutic evaluations [41]. CHC subjects with advanced fibrosis as defined by TE are younger in age and have a higher BMI, higher platelet counts, lower FIB-4 levels, higher incidence of fatty liver and splenomegaly, and higher CAP values than those identified by FIB-4 [42].

Inter-operator variability during TE has, however, also led to controversy regarding its objectivity in detecting advanced fibrosis. Several studies have been carried out, including a cross-sectional study, in which no significant difference in fibrosis assessment was found between two operators performing 195 procedures [43]. Often, non-fasting can influence LS measurement. Arena et al. [44] conducted work which aimed to investigate LS-enhancing factors during fibrosis assessment in 125 patients with CHC. The LS values were obtained after overnight fasting and 15, 30, 45, 60, and 120 min after the intake of a standardized liquid meal (400 mL, 600 Kcal, 16.7% of proteins, 53.8% of carbohydrates and 29.5% of fats). A large increase in LS was observed from 15 to 45 min after the meal, with the return of LS values to baseline within 120 min. It is probable that changes in LS values after a meal are related to hepatic microvascular readjustment, leading to increased portal blood flow, which is in agreement with postprandial hyperemia associated with increased portal pressure in cirrhotic patients [45]. The increase in postprandial LS values together with the fibrotic evolution of CHC could represent an indirect index of the progressive deterioration of the vascular autoregulatory mechanisms, in particular of the hepatic sinus circulation as a consequence of inflammation, fibrotic remodeling, and neoangiogenesis [46,47]. For the non-invasive assessment of fibrosis, an additional score based on the platelet count, serum type III procollagen peptide, type IV collagen 7S domain, central triple-helix of type IV collagen, and tissue inhibitor of metalloproteinases has been proposed. The rationale for this is based on the observation of decreased platelet counts and increased type IV collagen 7S levels in patients with CHC cirrhosis. Type IV collagen 7S forms, indeed, the basement membrane of the hepatic sinus and its production is enhanced with basement membrane hyperplasia and fibrosis progression [48]. However, hyaluronic acid is produced mainly by fibroblasts, stellate cells, and synovial cells, and is related not only to fibrosis but also to inflammation [49]. This new formula produced scores that were highly correlated with LS and showed a better performance than conventional fibrosis indices. However, more studies are needed to confirm its utility in detecting patients with cirrhosis, particularly those at a high risk of hepatocellular carcinoma after treatment with DAAs for HCV eradication [50]. Given its advantages over invasive methodologies, TE can help to better delineate the diagnostic assessment of CHC progression [51]. Table 1 lists the main papers on the topic.

## 4. Changes in LS in Patients Undergoing Antiviral Therapy

The introduction of antiviral drugs has revolutionized hepatology and, above all, the prognosis and quality of life of patients [52,53]. With regard to HCV, clearance occurs in almost all patients. This results in the inhibition of inflammatory activity and cyto-necrosis and in at least the partial regression of fibrosis [54,55]. Although traces of HBV may still persist in the body, it is in fact poorly active. In patients with cirrhosis, where it is not possible to obtain a *restitutio ad integrum* of the tissue, episodes of decompensation cease, and a longer life expectancy is observed [56,57,58]. The impact of these therapies on fibrosis regression has been also assessed by FibroScan^®^ [59,60]. Although liver biopsy is the optimal procedure for tissue evaluation, it is difficult to propose an invasive assessment to patients with a long-standing absence of viremia and cytonecrosis. A recent retrospective study evaluated 465 HBV patients treated with lamivudine (median baseline LS 8.7 KpA) and tenofovir (median LS baseline 11.7 KpA) and who were monitored for at least one year after the end of therapy. The mean decline in LS was greater in the tenofovir group (−4.2 KpA, median LS 7.0 KpA) than in the lamivudine group (−1.6 KpA, median LS 6.7 KpA), and was paralleled by a more effective control of hepatic cytolysis and a greater HBV negativization rate in the tenofovir group (90%) [61]. Similarly, another recent retrospective study evaluated the LS dynamics in HBV patients who were treated with nucleos(t)ide analogues (NAs) and monitored annually for 64 months. The decrease in LS was greater in patients with initial cirrhosis after the first year and in patients with higher transaminase levels. In addition, HBV-DNA became undetectable in 90% of the population by the third year [62]. A study by our group also found a greater decrease in LS after treatment with NAs in patients with higher baseline values (−6 kPa in cirrhosis-advanced fibrosis with 14.1 KpA at baseline and −1.6 in nonadvanced fibrosis with 6.9 KpA at baseline) [63]. Seto et al. found a greater decline in LS in patients with no metabolic risk factors, especially those with a BMI ≤25. Furthermore, HBV was undetectable in 97% of patients for a median of 87 months [64]. LS reductions in treated HBV patients were observed in different studies regardless of the chosen drug (entecavir or tenofovir) and were accompanied in most cases by aminotransferase normalization and HBV-DNA disappearance [65,66,67]. However, the main limitation of these studies was the lack of histological evaluation after the end of therapy. LS decline was, therefore, considered as suggestive of fibrosis regression.

Some studies have assessed a second biopsy after NA therapy. Marcellin et al. evaluated 348 HBV patients with liver biopsies at the initiation of tenofovir therapy and at 240 months after discontinuation. Overall, 87% of the patients showed histological improvement (≥2 point reduction in Knodell necroinflammatory score) and 51% displayed fibrosis regression (≥1 unit decrease by Ishak scoring system). Interestingly, 74% of cirrhotic patients (Ishak score 5 or 6) showed cirrhosis regression [68]. Sun et al. recently performed TE and liver biopsy 96 weeks after the discontinuation of entecavir in a population of 148 HBV patients. The LS decline was paralleled by histological improvement (area under the ROC curve [AUROC] 0.70), thus confirming the usefulness of FibroScan^®^ for the long-term monitoring of HBV patients [69].

Concerning the HCV setting, LS regression has been shown to be fast after a response to DAAs is observed [11,60,70]. Bachofner et al. evaluated 392 patients within 18 months of the end of treatment (EOT); there was a 32% decline in LS (median 12.6 KpA baseline, 8.5 KpA) in SVR patients accompanied by a reduction in APRI and FIB-4 scores [71]. These data were confirmed by Sporea et al., who showed a decline in LS of approximately 60% at the end of treatment and 75% at 12 weeks after EOT in a population of cirrhotic patients who achieved a SVR [72]. Knop et al. evaluated a cohort of 260 patients who achieved a SVR at EOT and at 24 months. Significant improvements in LS (median 8.7 KpA baseline, 7.9 KpA at 24 months), with a greater reduction in patients classified as F4 (25 Kpa baseline, 21.5 at 24 months), were reported. The significant reduction in LS correlated with the normalization of ALT, while cirrhotic patients with thrombocytopenia did not demonstrate a decline in LS [73]. Conversely, a recent retrospective study showed slightly different results. The LS was longitudinally assessed in an overall HCV population of 813 patients divided into two groups: one treated with DAAs (52%) and the other untreated. At least two LS measurements were performed for each group at a mean time point of 11.7 and 12.7 months, respectively. The authors showed higher LS values at baseline to be best associated with significant LS decline; moreover, DAA treatment was not associated with significant changes in liver stiffness over time in the two groups [74]. 

LS reduction in the years following SVR has also been studied in relation to death and other complications of chronic liver disease. In a study on 456 patients with a baseline LS of ≥10 KpA and with their SVR monitored for at least 24 months, a reduction of ≥20% was associated with a lower risk of death or decompensation, encephalopathy, and varices [75]. A study carried out by our group (516 HCV patients, with 301 cirrhosis) found a decrease in LS over time, which was faster in the initial phase after treatment and slower but constant in the monitoring phase for up to 24 months. However, patients who developed HCC or who had ultrasound-detected steatosis did not show a late reduction in LS [76]. No CAP software was available for this study at the time. Subsequent studies have focused on steatosis in the long-term monitoring of treated patients. Rout et al. prospectively included 372 patients with chronic HCV disease (25% cirrhosis) in an assessment of LS and CAP 12 weeks and 1 year after EOT. In addition to the decline in LS that was already observed in other studies, they showed a significant increase in CAP (median 25 d/m) in 66% of patients [77]. This increase was not associated with weight gain or alcohol consumption, and a clear explanation was not provided. Similarly, another study found an increase in CAP 72 weeks after EOT in a cohort of 214 treated HCV patients, which correlated with an increase in cholesterol. Additionally, a significant LS reduction was reported [78]. 

The LS reduction observed in most studies was not supported by histological data showing fibrosis regression. There are no recent, large studies including liver biopsy after DAA-induced SVR; the few available data do not show a total correspondence between ΔLS and fibrosis regression [79,80]. The rapid reduction in LS is certainly attributable to the abolition of cytolytic activity; this is followed by an improvement in the fibrotic status that could be assessed with a long-term measurement of LS and serum fibrosis markers.

## 5. LS in Portal Hypertension

Portal hypertension (PH) is one of the main complications of liver cirrhosis and a prerequisite for the development of ascites, varices, and hepatic encephalopathy [81]. Varicose vein bleeding and hepatic coma are some of the determinants of mortality in decompensated cirrhotic patients [82,83]. The gold standard for PH measurement is hepatic venous pressure gradient (HVPG), with a clinically significant portal hypertension (CSPH) set at a cut-off of >10 mmHg [84]. However, since HVPG is only available in a few centres with dedicated, highly specialized personnel, FibroScan^®^ has been tried in routine clinical practice as a possible non-invasive method. A recent meta-analysis of 26 studies which included 4337 patients compared TE and HPVG. A cut-off of 22.8 kPa was shown to correlate with CSPH diagnosis (HPVG > 10) with good sensitivity and specificity [85]. In another meta-analysis, the ability of LS to detect esophageal varices was investigated. Unfortunately, LS was not deemed accurate in differentiating the sizes of the varices in liver diseases due to them having different causes [86]. A recent consensus conference proposed the term compensated advanced liver disease (CALD) based on LS values to stratify patients at risk of CSPH regardless of their histology [87]. Values < 10 kPa would exclude CALD, while values >15 kPa may be considered highly suggestive. In addition, a <15 kPa value plus platelet count >150,000 reliably rules out CSPH (sensitivity and negative predictive value > 90%). Conversely, virus-, alcohol-, or NASH-related CALD patients with a LS >25 kPa are considered as having CSPH (specificity and positive predictive value of 90%). Concerning the grey area between 15 and 25 kPa, the consensus assessed the risk of CSPH according to the ANTICIPATE model. Patients with LSM values between 20 and 25 kPa and a platelet count <150,000, or LSM values between 15 and 20 kPa and a platelet count <110,000, have a CSPH risk of at least 60%. In addition, patients who cannot implement prophylaxis with β-blockers should undergo endoscopy if they have a LS 20 kPa or PLT <150,000. LS changes in CALD patients undergoing treatment have also been evaluated. For example, HCV patients who have achieved SVR and whose LS drops below 12 kPa with a platelet count >150,000 can be considered no longer at risk of CSPH. A LS value of <20 kPa plus a platelet count >150,000 in HCV and HBV patients who have achieved SVR can be used to rule out high-risk varicose veins. Overall, the Baveno VII consensus considers FibroScan^®^ to be accurate in identifying CSPH [87].

A recent international study evaluated 1159 CALD patients, 36.8% with CSPH, to assess the ability of non-invasive methods to predict the risk of decompensation. The non-invasive assessment of CSPH predicted the risk of decompensation with high accuracy, while no decompensation was observed in the case that CSPH was ruled out. Concerning the grey area, the risk of decompensation proved negligible in virally induced CALD patients, while it was higher in the nonviral CALD group. In addition, the study highlighted the usefulness of preventive nonselective β-blocker treatment for varicose veins in CSPH patients [88]. 

The performance of non-invasive tests, including the von Willebrand factor antigen to PLT ratio (VITRO), was evaluated in a cohort of 301 CALD patients with HPVG >10 mmHg. The application of the Baveno VII-VITRO algorithm improved the identification of CSPH patients by relocating the patients that were not classified according only to Baveno VII only (45.6% of the cohort) [89]. Similarly, Dajti et al. retrospectively evaluated a cohort of 140 patients with no episodes of decompensation who were undergoing HPVG, LS, and spleen stiffness assessment. The LS grey zone for CSPH (40–60% of the sample) was reduced to 7–15% by adding the cut-off of 40 kPa for spleen stiffness to the algorithm, with an adequate positive and negative predictive value [90].

A recent European study which aimed to identify compensated advanced chronic liver disease (cCALD) evaluated 3606 patients with TE and 1772 patients who had undergone a liver biopsy. Cut-offs of <8 (<7 for viral hepatitis) and >12 were reported as those with the highest sensitivity and specificity (91 and 92%, respectively) for cACLD [91].

In conclusion, LS is a reliable procedure for identifying or ruling out CSPH. For intermediate values, adding coagulation and splenic stiffness variables may further enhance the selection of high-risk patients.

## 6. LS in NAFLD

Non-alcoholic fatty liver disease (NAFLD) has become the most common liver disease worldwide, with a global prevalence of 25% [92]. It is characterized by fat accumulation in more than 5% of hepatocytes, which can lead to inflammation, fibrosis, cirrhosis, and the development of hepatocellular carcinoma (HCC). According to the guidelines issued by the EASL, the American Association for the Study of Liver Diseases (AASLD) and the Japanese Society of Gastroenterology, the gold standard for the diagnosis and evaluation of steatosis and fibrosis in NAFLD is liver biopsy [93,94,95]. However, it is an invasive procedure that is not without complications, and has bias due to the expertise of the operator and the heterogeneity of liver tissue. Moreover, it is not always well accepted by the patient [96]. VCTE by FibroScan^®^ is a non-invasive method that is able to evaluate hepatic fibrosis by LSM and to quantify liver fat by the controller attenuation parameter (CAP), with a specificity of 91% and a sensitivity of 87% for hepatic steatosis detection [97]. It was also the first available non-invasive method and the best-validated method in multicentre trials and meta-analyses [98]. It is recommended in the current NAFLD clinical practice guidelines from the EASL, the European Association for the Study of Diabetes (EASD), and the European Association for the Study of Obesity (EASO) as a non-invasive procedure for the evaluation of liver fibrosis in patients with NAFLD [99]. LSMs range from 1.5 kPa to 75 kPa, with higher values indicating more severe fibrosis; NAFLD fibrosis cut-off values, according to published VCTE LSMs, are as follows: F0–F1, <7.9 kPa; F2, from 7.9 to <8.8 kPa; F3, from 8.8 to <11.7 kPa; and F4, ≥ 11.7 kPa (Table 2) [100]. FibroScan^®^ has very good sensitivity and specificity for diagnosing fibrosis in patients with NAFLD [101]. According to Ozercan et al., VTCE has a sensitivity of 95% and a specificity of 77% in detecting liver fibrosis in patients with NAFLD. If a cut-off value of 9.9 kPa is considered for advanced fibrosis, AUROC is 0.93 (95% CI, 0.86–0.96) [102]. Thus, FibroScan^®^ makes it possible to avoid liver biopsy in at least 45% of patients [103]. Moreover, several studies have reported the AUROCs for detecting fibrosis stages (F ≥ 1 0.78–0.97, F ≥ 2 0.77–0.99, F ≥ 3 0.73–1.00, and F4 0.89–0.997), with an increase in sensitivity and specificity as the level of fibrosis increases [104]. Regarding the use of the M and XL probes, Oeda et al. showed no differences in accuracy in the AUROCs between the two probes [105]. Several factors may influence the assessment of liver fibrosis by FibroScan^®^ in patients with NAFLD, although studies related to patients with NAFLD are still scarce. First, LSM should be carried out after overnight fasting, or at least 2 h after a meal. This is because food intake can lead to an inappropriate increase in the LSM value [106,107,108]. As reported by Muller et al., an increase of 1 mg bilirubin can cause a 1 kPa increase in the LSM, and a 2 cm increase in intrahepatic venous pressure can increase the LSM by 1 kPa. An increase of approximately 100 U/L in aspartate aminotransferase (AST) can eventually cause an increase of 4 kPa in the LSM [109]. Finally, the degree of liver inflammation may impair the measurement of fibrosis by LSM; however, liver steatosis does not seem to affect the assessment of the degree of fibrosis [110]. 

Several factors may be associated with fibrosis in patients with NAFLD. In particular, hypertension (adjusted OR = 1.50, 95% CI: 1.09–2.08), high waist circumstance (adjusted OR = 2.61, 95% CI: 1.17–5.82), diabetes (adjusted OR = 3.97, 95% CI: 2.50–6.29), and metabolic syndrome (adjusted OR = 2.39, 95% CI: 1.83–3.12) have all been independently associated with severe fibrosis [111]. The degree of fibrosis, mainly in the cases of F3 and F4, as well as its variations, are the main drivers of prognosis in patients with NAFLD and are important risk factors for both liver and systemic complications [112,113,114]. Petta et al. proposed an algorithm for the risk stratification of complications in patients with NAFLD and cACLD, including LSM variation over time during patient follow-up. A variation of ˂20% relative to baseline LSM was associated with a low risk of complications; a variation between −20% and +20% was associated with an intermediate risk; and a variation of ˃20% was associated with a high risk of complications, including liver decompensation, HCC development, and death. Particularly, a baseline level ˃21 kPa was independently associated with a high risk of complications [115]. 

The evaluation of fibrosis by VTCE may also play a role in early liver disease screening. Eskridge et al. showed that, in patients with risk factors for the development of NAFLD, the early assessment of LSM, which is both feasible and acceptable for most patients, was able to encourage early therapeutic interventions with a significant improvement in prognosis [116].

Recently, the scientific community has proposed the MAFLD (metabolic associated fatty liver disease) acronym to more accurately reflect the pathogenesis and help with patient stratification for management [117]. However, since new studies based on MAFLD concepts are ongoing, no changes have been recommended yet in the application and interpretation of TE.

## 7. LS in Type 2 Diabetes Mellitus

The coexistence of NAFLD and type II diabetes mellitus (T2DM) not only increases the risk of developing the most severe histological forms of NAFLD, such as non-alcoholic steatohepatitis (NASH), cirrhosis, and hepatocellular carcinoma, but also increases the risk of developing chronic complications of T2DM [118,119]. The gold standard for the diagnosis of NAFLD and its evolution still remains liver biopsy. TE has increasingly emerged as a non-invasive method for the evaluation of liver damage and, in particular, the evaluation of LS and CAP through FibroScan^®^ [120,121]. LS and CAP are reliable measures not only for T2DM evaluation, but also for its complications. In fact, over the last few years, observational studies have found that advanced liver fibrosis is associated with major cardiovascular events and chronic kidney disease in patients with diabetes [122,123]. A recent multicentre, cross-sectional study [124] evaluated 442 outpatients with T2DM who were undergoing TE. One fourth of the cohort had a history of myocardial infarction or stroke, and half had microvascular complications. The prevalence of fatty liver disease (CAP 238 dB/m) and significant liver fibrosis (LSM 7.0/6.2 kPa) were 84.2% and 46.6%, respectively. Significant liver fibrosis was also associated with an increased likelihood of suffering from a heart attack, peripheral polyneuropathy, chronic kidney disease (CKD), or retinopathy independently of cardiometabolic risk factors, T2DM-related variables, and other confounders. However, hepatic steatosis was not independently associated with micro- and macro-vascular complications. Only one other cross-sectional study [125] demonstrated a higher risk of developing chronic vascular complications for patients with liver fibrosis and T2DM. Furthermore, a close association between an increased LSM (>7 kPa) and the presence of carotid atherosclerotic plaques was recorded, further supporting the increased risk of vascular complications in T2DM patients [126].

Several pathophysiological mechanisms are deemed to underlie the correlations among vascular damage, liver fibrosis, and diabetes. Among these, insulin resistance, which is the main promoter of atherogenic dyslipidemia and proinflammatory mediator synthesis, plays a major role [127]. Further studies have shown hepatic fibrosis to be an important risk factor, as opposed to steatosis, for cardiovascular complications and mortality in patients with T2DM and NAFLD [128]. The protective role of steatosis emerged from the evaluation of some genetic polymorphisms that are related to steatosis, such as single nucleotide polymorphisms (SNPs) in the PNPLA3 and TM6SF2 genes [129]. Polymorphisms of the TM6SF2 gene may be associated with reductions in LDL cholesterol and triglyceride serum levels, with consequent cardiovascular risk attenuation [130]. However, this was not found to be true for PNPLA3 [131]. 

Regarding the progression of hepatic fibrosis during follow-up in patients with diabetes, the patients’ LSM values increased over time from <10 kPa to >10 kPa, and this increase was shown to be closely related to increases in BMI, HbA1c, ALT, and ΔALT. An increase in patients’ CAP values from <248 dB/m to ≥248 dB/m was correlated with shorter T2DM duration, high BMI, low HDL cholesterol blood levels, high triglyceride levels, and high AST and ALT levels. However, various multivariate analyses revealed that only a high level of ALT can be considered an independent predictor of CAP increase [132]. Further studies which included liver biopsy have shown that changes in BMI and waist circumference are predictors of fibrosis progression over three years [133]. Given the high prevalence of T2DM in the global population, screening for NAFLD should ideally also take place in general practice. The American Diabetes Association (ADA) guidelines [134], in fact, recommend assessing NAFLD at the time of diagnosis of T2DM or prediabetes, with yearly revaluations. Only patients showing liver laboratory and imaging alterations should be referred to a hepatologist. Other international organizations, such as the EASL, recommend a non-invasive assessment of fibrosis in patients who are deemed to be at a high risk for NAFLD, including those with diabetes, regardless of liver enzyme elevations [135]. 

A new challenge is to now develop TE-based scores for liver fibrosis to stratify the risk in patients with T2DM. The diabetes fibrosis score (DFS) is calculated using the BMI, blood levels of platelets, AST, LDL and HDL cholesterol, and albuminuria [136]. DFS has been shown to be better than APRI [137], NFS, or FIB4 [138] for identifying advanced fibrosis in patients with NAFLD and T2DM [139]. The latest Asia Pacific Working Party on NAFLD Guideline recommends that NAFLD patients also make lifestyle modifications, such as greater-than-10% weight loss, as this has been shown to resolve NASH and improve liver fibrosis [140]. Several new drugs used for the treatment of T2DM have also proven indispensable for NASH treatment. For example, liraglutide, a GLP-1 agonist, has been shown to be useful in steatosis reduction and hepatocyte ballooning reversal, with minor progression towards liver fibrosis [141]. Empagliflozin, a SGLT2 inhibitor, also impacts both weight loss and liver damage [142]. A recent study evaluated the effects of dapagliflozin on fatty liver disease and fibrosis in patients with NAFLD using FibroScan^®^. At 24 weeks, patients treated with dapagliflozin, another drug belonging to the SGLT2 inhibitor family, achieved significant decreases in CAP (from 314 ± 61 dB/m to 290 ± 73 dB/m, *p* = 0.042), HbA1c, HOMA-IR, AST, ALT, and γ-GT compared to the control group. SGLT2 inhibitors have been shown to improve fibrosis of a moderate/severe degree [143]. Empagliflozin was able to reduce hepatic fat deposits as assessed by MRI-derived proton density fat fraction [144]. Research is still ongoing to elucidate the mechanisms by which SGLT2 inhibitors dampen liver injury [145,146].

The assessment of steatosis and fibrosis in patients with T2DM through non-invasive methods, such as TE by FibroScan^®^, allows for the recognition of patients with NAFLD, dictates the proper follow-up, and improves therapeutic strategies through the use of the new drugs for T2DM. Thus, the development of liver cirrhosis and/or hepatocellular carcinoma may be prevented in a patient population that is otherwise at a high risk [147].

## 8. LS in Autoimmune Liver Diseases

Primary biliary cholangitis (PBC), autoimmune hepatitis (AIH), and primary sclerosing cholangitis (PSC) are chronic autoimmune liver diseases (AILDs) that are generally distinguishable on the basis of their clinical manifestations but can sometimes manifest as overlapping conditions [148,149,150,151]. They are all characterized by the possible development of liver fibrosis or cirrhosis. Again, the gold standard for diagnosis and staging remains liver biopsy. However, the measurement of LS by VCTE performs remarkably well when compared to other non-invasive biomarkers of liver fibrosis. LSM appears to work better in PBC than in AIH, PSC, or overlapping syndromes. The diagnostic accuracy for liver fibrosis detection in PBC shows an AUROC of 0.94, 0.92, and 0.93 for significant fibrosis (SF), advanced fibrosis (AF), and cirrhosis, respectively, whereas, in AIH, moderate to excellent accuracy (with AUROC of 0.83, 0.91, and 0.90, respectively) has been reported [152].

### 8.1. PSC and LS

Primary sclerosing cholangitis (PSC) is a rare liver disease of unknown aetiology that is characterized by progressive sclerosis and the obstruction of large and small intra- and extra-hepatic bile ducts [153]. Chronic cholestasis and intermittent cholangitis are the main clinical features. PSC shows a variable course and evolution, which ultimately results in biliary cirrhosis [154]. The gold standard for disease diagnosis and staging is still endoscopic retrograde cholangiopancreatography (ERCP); however, magnetic resonance cholangiopancreatography (MRCP) is now recognized as a first-level non-invasive imaging method [155,156]. Several studies have attempted to demonstrate a correlation between LS measured with VTCE and the stenosis of intra- and extra-hepatic ducts as well as the degree of fibrosis. Tafur et al. and Corpechot et al. did not observe a statistically significant correlation between the severity of intrahepatic and extrahepatic stenosis and LS measured by VCTE, nor a correlation between LS measured by VCTE and either Mayo Risk Score or Amsterdam–Oxford prognostic index (AOPI). The only independent parameter associated with LSM was the degree of fibrosis. The cut-offs used by Tafur et al. and Corpechot et al. to classify the severity of mild to moderate and moderate to severe fibrosis were as follows: F0—F1/F2: <11.1 kPa; F2/F3—F4: ≥11.1 kPa [157,158].

### 8.2. AIH and LS

Autoimmune hepatitis (AIH) is a chronic immune-mediated liver disease characterized by the presence of interface hepatitis and plasma cell infiltration on histological examination, increased aminotransferase values, the presence of non-organ-specific autoantibodies in the circulation, and increased IgG levels. Chronic inflammation in the liver can evolve into fibrosis or even cirrhosis [159,160]. Diagnosis is based on a combination of serum markers and the presence of autoantibodies. Liver biopsy is still the gold standard for the diagnosis and evaluation of disease severity for AIH, including the stage of the fibrosis and the degree of inflammatory activity [161]. Biochemical remission is the primary treatment goal of AIH and is strongly associated with a reduction in LS as measured by VCTE. Several studies have attempted to demonstrate the utility of LSM as a non-invasive marker of fibrosis in the follow-up of AIH patients and to assess its performance as compared with biochemical markers (FIB-4, APRI). Xu et al. reported that LSM was strongly associated with the degree of fibrosis (r = 0.752, *p* < 0.01), with LSM values for stages F0, F1, F2, F3, and F4 being 4.3 ± 1.0 kPa, 5.9 ± 2.4 kPa, 7.3 ± 2.4 kPa, 11.9 ± 6.9 kPa, and 20.2 ± 9.8 kPa, respectively. The AUROC values of LSM in detecting fibrosis stages F ≥ 2, F ≥ 3, and F4 were 0.878, 0.883, and 0.914, respectively. The optimal LSM cut-off values for the liver fibrosis stages were 6.45 kPa for ≥F2, 8.75 kPa for ≥F3, and 12.50 kPa for F4. Furthermore, the study showed the superiority of LSM over the APRI score and FIB-4 in detecting severe fibrosis (F ≥ 3) [162] (Table 3). Hartl et al. showed that complete biochemical remission was strongly correlated with LS reduction (“remission”: −7.5%/year vs. “no remission”: +1.7%/year, *p* < 0.001), suggesting fibrosis downgrading. However, LS changes were dependent on the degree of fibrosis. No significant variations were found for patients with F0–1 fibrosis (−0.5%/year; 95% CI −5.0% to 4.1%; *p* = 0.8), whereas a significant LS reduction was found in patients with advanced fibrosis (F2: −5.1%/year; 95% CI −9.9 to −0.7%; *p* = 0.025; F3: −8.3%/year; 95% CI −13.0 to −2.9%; *p* = 0.002) and, particularly, in cirrhotic patients (F4: −11.7%/year; 95% CI −19.1% to −3.5%; *p* = 0.006). Overall, these findings support the reversibility of severe fibrosis in AIH patients who achieve a complete response to treatment [163].

### 8.3. PBC and LS

Primary biliary cirrhosis is a chronic autoimmune liver disease which results from the progressive destruction of hepatic bile ducts by circulating autoantibodies [164]. Liver biopsy remains the gold standard for its diagnosis (along with the detection of serum autoantibodies and high serum levels of alkaline phosphatase [ALP]) and staging. Recently, non-invasive biochemical markers, including the mac-2 binding protein glycosylation isomer (M2BPGi), FIB-4, and APRI, have been used for the assessment of the disease progression and fibrosis degree [165]. LSM using VCTE is recommended by the EASL for the staging and follow-up of PBC [166]. Several studies have made attempts to define cut-offs for the detection and staging of fibrosis. Osman et al. identified optimal LS cut-off values for predicting the presence of fibrosis concordant with histological assessment; these values were 6.60 kPa (AUC 0.70), 7.00 kPa (AUC 0.65), 7.50 kPa (AUC 0.73), and 14.40 kPa (AUC 0.94) for stages ≥F1, ≥F2, ≥F3, and F4, respectively. Furthermore, the same authors identified the value of 10.20 kPa (HR, 13.73; 95% CI, 2.77–68.06) as the cut-off to predict hepatic decompensation [167] (Table 3). Conversely, Corpechot et al. [168] identified the values of 8 kPa and 15 kPa (*p* < 0.0001) as optimal cut-offs for LSM to categorize patients into the categories of low, medium, and high risk at the first measurement. They also suggested that baseline values >9.6 kPa could help to identify patients at a higher risk of an unfavourable outcome. Moreover, the association between LSM and the Globe score (with cut-offs of 0.5 and 1.8 for division into the three different risk groups) was shown to increase the accuracy of risk stratification. Finally, LSM was shown to be significantly associated with the long-term outcome in patients with PBC, performing even better than ALP and bilirubin values. Thus, these findings suggest the possibility of using LSM as a surrogate survival endpoint. Moreover, LSM may be useful to monitor patients responding to ursodeoxycholic acid (UDCA), as a reduction in LS may be expected to occur over time, or to detect the progression of fibrosis in UDCA-resistant subjects [168,169]. Cristoferi et al. identified 6.5 kPa as the optimal cut-off value to exclude or confirm the presence of cirrhosis in patients with PBC, regardless of the patient’s BMI [170].

**Table 3:** Xu [162] and Osman [167] cut-offs.

Stage	Aih Cut-Off (kpa)	Aih Auroc	Pbc Cut-Off (kPa)	Pbc Auroc
F0				
≥F1			6.60	0.70
≥F2	6.45	0.878	7.00	0.65
≥F3	8.75	0.883	7.50	0.73
≥F4	12.5	0.914	14.40	0.94

## 9. TE in Liver Transplant Recipients

Liver transplant recipients (LTRs) constitute a significant population of the patients evaluated in outpatient hepatology clinics. Remarkable progress in the selection of transplant candidates and the optimization of immunosuppressive therapy allows for a durable good quality of life in many cases [171,172,173,174,175].

In recent years, metabolic or alcohol-based cirrhosis has progressively replaced virally induced cirrhosis as a reason for liver transplantation [176]. Consequently, the long-term problems that are expected during post-transplant life have also changed. The administration of protective antibodies against HBV and HCV eradication with new therapies has almost completely eliminated problematic reinfection [177]. While metabolic problems remain to be prevented, the appearance of steatosis and fibrosis, and chronic rejection, still occurs [178,179,180]. The role of FibroScan^®^ in this category of patients has been tested by many studies in the pre- and post-transplant phase [181]. The pilot study by Mancia [182] compared the accuracy of FibroScan^®^ versus liver biopsy in the liver of a deceased donor, with satisfactory results for fibrosis (75%) and for fibrosis and steatosis with the use of CAP (95%). Other studies have evaluated the performance of FibroScan^®^ in living donors, with mixed results [183,184,185]. In these cases, in fact, the need to evaluate the patient from a biliary and vascular surgical perspective requires a traditional radiological assessment (computed tomography/MRI). 

Some studies have evaluated the usefulness of FibroScan^®^ in acute rejection. Crespo et al. [186] suggested a cut-off of 8.5 KpA for moderate to severe rejection, and found a positive predictive value of 100%. Similarly, Rigamonti et al. [187] identified 7.4 KpA as the 100% specific cut-off for graft damage. LS increase in acute rejection is mediated by the inflammatory process; therefore, TE offers a great advantage in suggesting this clinical occurrence. Unfortunately, only a few studies have compared TE and liver biopsy in acute rejection. Furthermore, a hepatology team cannot rely exclusively on the physical data of LS, as the detailed information provided by histology is pivotal to selecting an immunosuppressive therapy [181,188]. Conversely, the onset and progression of fibrosis are frequent events during the years following transplantation, and the periodic monitoring of LS may be of great diagnostic value in clinical practice. From this perspective, a recent meta-analysis reviewed a total of 24 studies in which the accuracy of TE was shown to be superior to that of both APRI and FIB-4, regardless of the cause of transplantation [189].

The occurrence of fibrosis or LS alterations related to chronic rejection has been investigated in different ways. For instance, in our experience, increased stiffness (21% of patients) was associated with both an HCV-RNA positive status and the presence of an active biliary complication of liver transplantation [190]. In a recent prospective study, the value of 10.5 kPa was recognized as the threshold for advanced fibrosis (F3 sec. Metavir), with an AUROC of 0.94 [191]. The increase in LS over time and the correlation with blood chemistry tests may help to select candidates for liver biopsy, as histological data may become fundamental in the decision-making process of LTRs. 

In the pre-DAA era, the evaluation of transplant patients for HCV cirrhosis was one of the main subjects of study. In a study where histological data were available, corresponding LS cut-off values of 5.6 KpA and 16.7 KpA were established for mild-to-moderate fibrosis and for cirrhosis, respectively [192]. Crespo et al. identified the LS value of 8.7 KpA as a highly accurate cut-off for the prediction of mortality and decompensation [193]. Mauro et al. evaluated LTRs that were treated with DAA and achieved SVRs. In these patients, the cut-off values of 10.6 and 14 kPa were able to rule out or diagnose cirrhosis, respectively; similarly, the values of 11.3 and 23 kPa could rule out or identify portal hypertension, respectively [194]. 

NAFLD may affect LTRs as well, either because of superimposed risk factors (overweight, diabetes, hyperlipidemia, hypertension) or due to de novo NAFLD, which has a prevalence of up to 26% [195]. In addition, the recurrence of steatohepatitis has been reported in up to one-third of NASH transplant patients [196,197]. A recent study evaluated 150 LTRs for steatosis and fibrosis with FibroScan^®^ equipped with CAP software [198]. Steatosis was found in 70% of the patients (with a severe form in 40%). In addition, liver biopsy performed on five patients with severe fibrosis as detected by TE showed the presence of chronic rejection. Since biochemical parameters were normal in some of the patients with steatosis or severe fibrosis, the importance of FibroScan^®^ in the clinical management of LTRs cannot be neglected. 

In conclusion, although TE may be useful in helping to detect inflammation and portal hypertension in LTRs, the lack of standardized cut-offs suggests a cautious approach towards the interpretation of results [199].

## 10. TE and Future Prospects

TE is an established procedure in clinical hepatology. Non-invasive measurement of fibrosis allows for strongly limiting the number of liver biopsies and to monitor a patient’s status over time. The limitations of the method are known but an experienced hepatologist can still implement a personalized diagnostic strategy to optimize the results. Currently, the need for histological data by liver biopsy has an important role in autoimmune diseases, as the type of liver damage directs both diagnosis and therapy [161]. In NAFLD, the guidelines suggest a liver biopsy when the results of non-invasive examinations (laboratory and instrumental) are controversial for significant fibrosis detection [99]. 

Fibroscan^©^ technology has made further progress in recent years. In fact, devices are now equipped with conventional ultrasound probes to detect the measuring point in the case of obese patients or those with a difficult anatomical conformation. This innovation allows for the use of FibroScan^©^ not necessarily in an environment where ultrasound is available. Moreover, the latest FibroScan^©^ models have the continuous CAP mode (c-CAP), which allows for quantifying steatosis independently from fibrosis measurement [200]. These latest generation models simply work by placing the probe at the hepatic surface without the aid of the elastic wave. This innovation makes the examination faster and more accurate, also considering that the device signals the operator if the probe is examining an adequate liver area. A possible non-invasive diagnostic alternative is multiparameter magnetic resonance imaging (MRI) elastography, which has a high diagnostic accuracy for both fibrosis and steatosis. MRI may be difficult to implement in daily clinical practice due to high costs and because it cannot be moved bedside; therefore, it is generally used in clinical studies as a non-histological gold standard [201]. A large meta-analysis comparing all non-invasive methods confirmed a higher diagnostic accuracy for MRI elastography [202]. Other non-invasive methods for measuring fibrosis include share wave elastography, which makes use of a dedicated software that is available on the latest generation of ultrasound devices. This technology is as reliable as FibroScan^©^, with the advantage of being used for liver diagnostics as a single device [10]. However, use of the ultrasonographic wave instead of the elastic wave impairs measurements in patients with meteorism or with thick adipose tissue. In our experience, the FibroScan^©^ XL probe may suffice in these settings. In the near future, Fibroscan^©^ will continue to play a central role in liver diagnostics. The development of a new software that is able to identify inflammation in the context of fibrosis is awaited to distinguish whether fibrosis or inflammation are responsible for a LS increase. This would eliminate a confounding factor and allow for the identification of NAFLD patients who are at greater risk of progression.

## 11. Conclusions

TE by FibroScan^®^ is widely used in clinical hepatology. Despite several drawbacks, which will be hopefully overcome in the near future due to technology advancements, this procedure may yield significant information in all aspects of clinical hepatology, optimizing the diagnosis and management of fibrosis and steatosis in different liver diseases, with limited resort to liver biopsy.

## Figures and Tables

**Table 1:** Main papers relating to CHB and CHC patients with LS cut-off values > F2.

Authors	Country	Type of Study	All Patients	HCV Patients	HBV Patients	Significant Fibrosis (≥f2)	Significant Fibrosis in HCV Patients	Significant Fibrosis in HBV Patients	*p* Value	Liver Stiffness	% Sensitivity	% Specificity
Ferraioli et al. [2]	Italy	Cross-sectional	246	195	41	117	97	11	0.004	>9 kPa	80	85
Friedrich-Rust et al. [3]	Germany	Meta-analysis	518	380	51	312	222	27	<0.0001	>7.65 kPa	79	85
Cardoso et al. [24]	France	Cross-sectional	565	363	202	282	197	85	<0.001	>7.2 kPa	74	88
Foucher et al. [26]	France	Prospective	711	398	43	243	-	-	<0.0001	>7.2 kPa	64	85
Chen et al. [36]	China	Prospective	375	-	291	231	-	231	<0.05	>9.8 kPa	82	94.5
Poynard et al. [30]	France	Prospective	1547	28	1433	-	-	206	<0.0001	>9.5 kPa	84	90
Castéra et al. [34]	France	Longitudinal	412	7	201	-	-	60	<0.0001	>7.2 kPa	87	80

**Table 2:** Diagnostic accuracy for liver fibrosis in patients with NAFLD.

Authors	Fibrosis Score	Lsm	Auroc
Ozercan et al. [102]	F3	>9.9 kPa	0.93
Oeda et al. [105]	F1	<7.9 kPa	0.78–0.97
	F2	7.9–8.8 kPa	0.77–0.99
	F3	8.8–11.7 kPa	0.73–1.00
	F4	>11.7 kPa	0.89–0.99

## Data Availability

Not applicable.
